# Local versus systemic effect of ovulation-inducing factor in the seminal plasma of alpacas

**DOI:** 10.1186/1477-7827-3-29

**Published:** 2005-07-14

**Authors:** Marcelo H Ratto, Wilfredo Huanca, Jaswant Singh, Gregg P Adams

**Affiliations:** 1 Veterinary Biomedical Sciences, Western College of Veterinary Medicine, University of Saskatchewan, S7N 5B4, Canada; 2 Faculty of Veterinary Medicine, San Marcos University, Lima, Peru

## Abstract

**Background:**

Camelids are induced (reflex) ovulators. We have recently documented the presence of an ovulation-inducing factor (OIF) in the seminal plasma of alpacas and llamas. The objective was to test the hypothesis that OIF exerts its effect via a systemic rather than a local route and that endometrial curettage will enhance the ovulatory response to intrauterine deposition of seminal plasma in alpacas.

**Methods:**

Female alpacas were assigned randomly to 6 groups (n = 15 to 17 per group) in a 2 × 3 factorial design to test the effect of seminal plasma versus phosphate-buffered saline (PBS) given by intramuscular injection, by intrauterine infusion, or by intrauterine infusion after endometrial curettage. Specifically, alpacas in the respective groups were given 1) 2 ml of alpaca seminal plasma intramuscularly, 2) 2 ml of PBS intramuscularly (negative control group), 3) 2 ml of alpaca seminal plasma by intrauterine infusion, 4) 2 ml of PBS by intrauterine infusion (negative control group), 5) 2 ml of alpaca seminal plasma by intrauterine infusion after endometrial curettage, or 6) 2 ml of PBS by intrauterine infusion after endometrial curettage (negative control group). The alpacas were examined by transrectal ultrasonography to detect ovulation and measure follicular and luteal diameters.

**Results:**

Intramuscular administration of seminal plasma resulted in a higher ovulation rate than intrauterine administration of seminal plasma (93% versus 41%; P < 0.01), while intrauterine seminal plasma after endometrial curettage was intermediate (67%). None of the saline-treated controls ovulated. The diameter of the CL after treatment-induced ovulation was not affected by the route of administration of seminal plasma.

**Conclusion:**

We conclude that 1) OIF in seminal plasma effects ovulation via a systemic rather than a local route, 2) disruption of the endometrial mucosa by curettage facilitated the absorption of OIF and increased the ovulatory effect of seminal plasma, and 3) ovulation in alpacas is not associated with a physical stimulation of the genital tract, and 4) the alpaca represents an excellent biological model to evaluate the bioactivity of OIF.

## Background

Early studies of South American camelids documented that copulatory stimulation is responsible for inducing ovulation in these species [1,2]. The first significant increase in plasma LH concentrations occurred 15–40 minutes after the initiation of mating in llamas and alpacas [3,4]. A similar LH increase was observed in Bactrian and dromedary camels (related camelid species) beginning 20–30 min after mating [5,6]. The rapid increase in plasma LH concentration after mating in camelids resembles that observed in rabbits [7] and cats [8] – also classified as induced ovulators. A 40-fold increase in GnRH secretion from the medio-basal hypothalamus was detected within 20–60 minutes of mating in rabbits [9], followed by a preovulatory LH surge and ultimately ovulation about 10 hours after mating [10].

The primary mechanism responsible for ovulation induction in these species is thought to involve a neuro-endocrine response to physical stimulation of the vagina and cervix by the penis during mating [11]. The results of recent studies in llamas and alpacas, however, provide support for the hypothesis that a chemical substance in the semen is responsible, in whole or in part, for inducing ovulation [12]. The existence of a potent ovulation-inducing factor (OIF) was demonstrated by intramuscular administration of cell-free llama and alpaca seminal plasma to females of the respective species. Collectively, 28 of 30 (93%) females ovulated after seminal plasma treatment compared to 0 of 32 (0%) saline-treated controls [12].

The discovery of OIF in llamas and alpacas is consistent with an early study in which intrauterine or intramuscular administration of Bactrian semen induced ovulation in Bactrian females [13,5]. However, conflicting results have been reported about the effect of local versus systemic administration of semen. In female alpacas that were mounted by a male (intromission prevented) and those that were mounted (intromission prevented) followed by artificial insemination, ovulation (detected at necropsy 3 days later) occurred in 2/15 and 3/9, respectively [1]. Since ovulation occurred in 36/44 females after natural copulation, the authors concluded that the physical act of coitus was responsible for eliciting ovulation in alpacas. In contrast, ovulation was detected in 6/10 alpacas and 5/8 llamas inseminated intravaginally with conspecific semen cited in [14]. In Bactrian camels, ovulation was detected by rectal palpation after intravaginal or intrauterine infusion of whole semen or seminal plasma in ≥ 75% of females [13,5,15]. In a recent ultrasonographic study [12], ovulation was detected in 13 of 14 alpacas given seminal plasma intramuscularly, but in 0 of 12 given seminal plasma by transcervical intrauterine deposition.

The reason for the disparity in results is not clear, but authors of the latter study [12] speculated that differences may be attributed to attenuated absorption of OIF from the genital mucosa compared to the muscle. In this regard, copulation in alpacas and llamas is a prolonged event (30 to 50 minutes) [16,3] and ejaculation is intrauterine [17]. A normal sequela of copulation in these species is acute, transient inflammation of the endometrium as a result of repeated abrasion by the penis [17]. Perhaps absorption of OIF in seminal plasma subsequent to natural mating is facilitated by the hyperemia of the excoriated endometrium.

The objective of the present study was to test the hypothesis that OIF exerts its effect via a systemic rather than a local route and that endometrial curettage will enhance the ovulatory response to intrauterine deposition of seminal plasma in alpacas. A 2-by-3 factorial design was used to compare the ovulatory effects of alpaca seminal plasma versus phosphate-buffered saline (control) administered by intramuscular injection, by intrauterine deposition, or by intrauterine deposition after endometrial curettage.

## Methods

### Seminal plasma collection

Semen was collected from male alpacas (n = 8) by artificial vagina [18] over a period of 2 months prior to the start of the experiment (10 ejaculates per animal) and processed as previously described [12]. Briefly, ejaculates were diluted 1:1 (v/v) with phosphate buffered saline (PBS, Gibco, Grand Island, N.Y., USA) and centrifuged for 30 minutes at 1500 × g. The supernatant was decanted to remove spermatozoa and a drop was evaluated by microscopy to confirm the absence of cells. If spermatozoa were detected, the sample was centrifuged again in like manner until all spermatozoa were removed. Sperm-free seminal plasma was stored at -70°C. Upon thawing, the diluted seminal plasma was pooled and kanamycin sulfate (Sigma Chemical Co., St Louis, MO, USA) was added to a final concentration of 25 μg/ml.

### Animals & Treatments

The study was conducted during February to March at the Quimsachata Research Station in the Department of Puno, Peru (15°S, 71°W, and 4,500 m above sea level) using mature non-lactating female alpacas ≥ 4 years of age and weighing an average of 75 kg. To facilitate data collection, ovarian follicular development was synchronized among females (n = 100) by giving 5 mg Armour Standard LH (Lutropin-V^®^, Bioniche Animal Health, Belleville, ON, Canada) to induce ovulation. We expected approximately 85% to 90% of the alpacas to ovulate after LH treatment, resulting in synchronous emergence of a new follicular wave 2 days after treatment [19]. Alpacas were examined by transrectal ultrasonography (Aloka 500 with a 7.5 MHz linear-array probe, Instruments for Science & Medicine Inc., Vancouver, BC, Canada) 12 days after LH treatment – sufficient time to permit complete luteal regression and growth of a new dominant follicle [19,20]. Alpacas with a follicle ≥ 8 mm in diameter (n = 92) were assigned randomly to 6 groups and given: 1) 2 ml of alpaca seminal plasma intramuscularly (n = 15), 2) 2 ml of PBS intramuscularly (control; n = 15), 3) 2 ml of alpaca seminal plasma by intrauterine infusion (n = 17), 4) 2 ml of PBS by intrauterine infusion (control; n = 15), 5) 2 ml of alpaca seminal plasma by intrauterine infusion after endometrial curettage (n = 15), or 6) 2 ml of PBS by intrauterine infusion after endometrial curettage (control; n = 15). Intramuscular injections were given in the semimembranosus muscle using a 20-gauge 40 mm long needle. Intrauterine infusions were accomplished by passing a plastic pipette through the cervix via transrectal manipulation and depositing 1 ml of alpaca seminal plasma or PBS into each uterine horn. To mimic the transient inflammation of the endometrium caused by the penis during natural mating [17], both uterine horns were curettaged before intrauterine infusion by repeatedly scraping the tip of the plastic infusion pipette back and forth over the surface of the endometrial of both uterine horns for 3 minutes. Curettage was accomplished by transrectal manipulation of the uterus with one hand and manipulation of the pipette with the other.

Alpacas were examined by transrectal ultrasonography on Day 2 (Day 0 = treatment) to detect ovulation. Ovulation was defined as the sudden disappearance of a large follicle (≥ 8 mm) that was detected during the previous examination [21]. To confirm ovulation and assess corpus luteum (CL) development, transrectal ultrasonography was repeated on Day 8; i.e., expected time of maximum CL diameter [21,20].

### Statistical Analyses

Single-point measurements (i.e., follicle size at the time of treatment, maximum CL diameter) were compared among groups by analyses of variance. If the overall effect was significant (P < 0.05), specific comparisons were made between groups using Tukey multiple comparisons. Ovulation rates were compared among groups by chi-square analysis.

## Results and Discussion

The diameter of the largest follicle at the time of treatment did not differ among groups (P = 0.9). Ovulations were observed in groups treated by intramuscular administration or intrauterine deposition of seminal plasma (Table 1). Ovulation and luteal development were not detected in females that were given PBS by intramuscular or intrauterine administration (control groups). The ovulation rate in the intramuscular group (93%) was higher (P < 0.01) than in the intrauterine group (41%), while the endometrial curettage group was intermediate (67%). Of the alpacas that ovulated, the diameter of the CL did not differ among groups.

**Table 1:** Effect of administration of alpaca seminal plasma administered intramuscularly or by intrauterine infusion with or without endometrial curettage on ovulation and corpus luteum formation (mean ± SEM) in female alpacas.

	Intramuscular		Intrauterine		Intrauterine with curettage	
	
	Seminal plasma	Phosphate buffered saline	Seminal plasma	Phosphate buffered saline	Seminal plasma	Phosphate buffered saline
Follicle diameter at treatment (mm)*	8.0 ± 0.3 (n = 15)	8.2 ± 0.3 (n = 15)	8.1 ± 0.3 (n = 17)	8.0 ± 0.3 (n = 15)	8.3 ± 0.2 (n = 15)	8.4 ± 0.3 (n = 15)
Ovulation rate (%)	14/15^a ^(93%)	0/15^c ^(0%)	7/17^b ^(41%)	0/15^c ^(0%)	10/15^ab ^(67%)	0/15^c ^(0%)
CL diameter (mm) on Day 8 (Day 0 = treatment)*	9.3 ± 0.4 (n = 14)	----	9.5 ± 0.3 (n = 7)	----	9.4 ± 0.4 (n = 10)	----

* No difference among groups (P ≥ 0.9)

^a,b,c ^Proportions with different superscripts are different (P < 0.01)

The results of the present study provide support for the hypothesis that the ovulation-inducing effect of seminal plasma is mediated via a systemic rather than a local route. A higher ovulation rate in alpacas treated by intrauterine infusion would have provided evidence to the contrary, but the ovulation rate was significantly lower in the intrauterine infusion group than in the intramuscular group. These results are consistent with those of a previous study [12] in which intramuscular administration of llama seminal plasma was followed by a surge in plasma LH concentration and ovulation. However, results do not unequivocally rule out a potential local contribution of seminal plasma to ovulation induction. In this regard, results of a study of the effects of boar seminal plasma deposited into different segments of the uterine horn in gilts were suggestive of a local unilateral mechanism influencing the interval to ovulation [22]. Ovulation was advanced in the ovary ipsilateral to the side of semen deposition, but interestingly, only when deposited near the utero-tubal junction; no effect was found when seminal plasma was deposited in the middle of the uterine horn between two ligatures. No information has been reported regarding circulating gonadotropin concentrations subsequent to intrauterine or intravaginal deposition of semen.

The results are also consistent with the concept that systemic absorption of OIF from the uterus is facilitated by endometrial curettage. The ovulation rate in the curettage group was intermediate between that of the intramuscular group and the intrauterine group without curettage. Endometrial curettage in the present study was mild and was accomplished by rubbing a smooth, round-tipped plastic infusion pipette against the endometrium for 3 minutes. Perhaps more aggressive curettage would induce sufficient endometrial inflammation to increase absorption of OIF and result in an ovulation rate more typical of natural mating during the period of follicular readiness (i.e., 90%; [23]).

The disparity between the present study and our previous study [12] in the effect of intrauterine treatment in non-curettaged alpacas (ovulation rate of 41% versus 0%, respectively) may be attributed to the dose and site of deposition of seminal plasma. A total of 2 ml of seminal plasma was infused in the uterine horns (1 ml in each horn) in the present study, while only 1 ml of seminal plasma was infused into the uterine body in the previous study [12]. Regarding local versus intramuscular absorption, the addition of a GnRH analogue (Buserelin) to the semen induced ovulation in rabbits after intravaginal artificial insemination [24], but the dose of GnRH required for ovulation induction by intravaginal deposition was ten times higher than that used by intramuscular administration in the control group (8 μg versus 0.8 μg per inseminated female). This is consistent with the results from our previous experiment [12] in which no ovulations were detected in alpacas after intrauterine deposition of 5 mg of LH (Lutropin), a dose that caused ovulation in more that 80% of the females when given intramuscularly [19,20]. Hence, higher systemic concentrations of OIF may have been achieved in the present study by using larger dose and causing greater dispersion of seminal plasma throughout the endometrial surface. No mention was made regarding uterine manipulations in previous studies in llamas and alpacas [1,14] or Bactrian camels [13,5,15], and it is unclear if semen was deposited into the vagina, the cervix, or the uterus.

Results did not support the notion that physical stimulation of the vagina, cervix and uterus is involved in a neuro-endocrine system for ovulation induction, nor was there any evidence that OIF is produced by tissues of the female reproductive tract. Despite purposeful manipulation and irritation of the genitalia in the present study, none of the 45 females treated with saline alone ovulated.

## Conclusion

We conclude that 1) OIF in seminal plasma effects ovulation via a systemic rather than a local route, 2) disruption of the endometrial mucosa by curettage facilitated the absorption of OIF and increased the ovulatory effect of seminal plasma, and 3) ovulation in alpacas is not associated with a physical stimulation of the genital tract, and 4) the alpaca represents an excellent biological model to evaluate the bioactivity of OIF.
